# Comparison of Persistent Symptoms After COVID-19 and Other Non-SARS-CoV-2 Infections in Children

**DOI:** 10.3389/fped.2021.752385

**Published:** 2021-10-29

**Authors:** Ieva Roge, Liene Smane, Anda Kivite-Urtane, Zanda Pucuka, Iveta Racko, Lizete Klavina, Jana Pavare

**Affiliations:** ^1^Department of Continuing Education, Riga Stradins University, Children's Clinical University Hospital, Riga, Latvia; ^2^Department of Pediatrics, Children's Clinical University Hospital, Riga Stradins University, Riga, Latvia; ^3^Department of Public Health and Epidemiology, Institute of Public Health, Riga Stradins University, Riga, Latvia

**Keywords:** COVID-19, SARS-CoV-2, pediatrics, clinical sequelae, viral

## Abstract

**Introduction:** The data on long COVID in children is scarce since children and adolescents are typically less severely affected by acute COVID-19. This study aimed to identify the long-term consequences of SARS-CoV-2 infection in children, and to compare the persistent symptom spectrum between COVID-19 and community-acquired infections of other etiologies.

**Methods:** This was an ambidirectional cohort study conducted at the Children's Clinical University Hospital in Latvia. The study population of pediatric COVID-19 patients and children with other non-SARS-CoV-2-community-acquired infections were invited to participate between July 1, 2020, and April 30, 2021.

**Results:** In total, 236 pediatric COVID-19 patients were enrolled in the study. Additionally, 142 comparison group patients were also enrolled. Median follow-up time from acute symptom onset was 73.5 days (IQR; 43–110 days) in the COVID-19 patient group and 69 days (IQR, 58–84 days) in the comparison group. Most pediatric COVID-19 survivors (70%, *N* = 152) reported at least one persistent symptom, but more than half of the patients (53%, *N* = 117) noted two or more long-lasting symptoms. The most commonly reported complaints among COVID-19 patients included persistent fatigue (25.2%), cognitive sequelae, such as irritability (24.3%), and mood changes (23.3%), as well as headaches (16.9%), rhinorrhea (16.1%), coughing (14.4%), and anosmia/dysgeusia (12.3%). In addition, 105 (44.5%) COVID patients had persistent symptoms after the 12-week cut-off point, with irritability (27.6%, *N* = 29), mood changes (26.7%, *N* = 28), and fatigue (19.2%, *N* = 20) being the most commonly reported ones. Differences in symptom spectrum among the various age groups were seen. Logistic regression analysis showed that long-term persistent symptoms as fever, fatigue, rhinorrhea, loss of taste and/or smell, headaches, cognitive sequelae, and nocturnal sweating were significantly associated with the COVID-19 experience when compared with the controls.

**Conclusions:** We found that at the time of interview almost three-quarters of children reported at least one persistent symptom, but the majority of patients (53%) had two or more concurrent symptoms. The comparison group's inclusion in the study allowed us to identify that symptom persistence is more apparent with COVID-19 than any other non-SARS-CoV-2 infection. More research is needed to distinguish the symptoms of long COVID from pandemic-associated complaints. Each persistent symptom is important in terms of child well-being during COVID-19 recovery.

## Introduction

Since March 2020, when the World Health Organization (WHO) declared a global pandemic due to the severe acute respiratory syndrome coronavirus 2 (SARS-CoV-2), an overwhelming volume of information related to coronavirus disease 2019 (COVID-19) has been published. At the beginning of the pandemic, only acute presentation of COVID-19 was described. In recent months, however, attention has been increasingly focused on its long-term consequences. Many COVID-19 patient advocacy groups, for those identifying themselves as “long-haulers,” have contributed to the recognition of post-acute COVID-19, and have forced the medical society to continue to expand the current research into the underlying pathophysiology, consequences, and rehabilitation options for affected patients.

According to the UK's National Institute for Health and Care Excellence's (NICE) suggested definition, “long COVID” is a general term describing the signs and symptoms which persist or develop after acute COVID-19, and it includes two sub-domains according to timeframe. Firstly, ongoing symptomatic COVID-19 is defined as symptoms persisting 4–12 weeks from the onset of the first acute symptoms. Secondly, if symptoms extend beyond 12 weeks, post-COVID-19 syndrome can be diagnosed (1, 2). Up to this point, there has been no consensus on a definition of long COVID, so timeframe, terminology, and classification can vary from one publication to another. It has been suggested that the definition could be adapted for different COVID-19 patient groups, taking into consideration length of hospitalization, predisposing intrinsic (age, gender, and comorbidities) and extrinsic (biological, psychological, and social) factors (2–4). At this point, there is no clear case-definition for long COVID among the pediatric population.

The precise prevalence of long COVID remains unknown, and the data is heterogeneous, ranging from 10 to 87% in adults to 4 to 66% in children (5–15). This wide range could be explained by lack of systematic, long-term follow-ups, with high variability in terms of age, severity of infection, prevalence of pre-existing clinical conditions, and characteristics of the clinical evaluation (since most pediatric studies are based on self- or parent-reported symptom persistence using questionnaires or online surveys) (7, 14, 16, 17).

While many publications are discussing long COVID in adults, the data on children is relatively scarce, since children and adolescents are generally less severely affected by acute COVID-19 than adults, leading to the false assumption that late-COVID-19 sequelae do not affect this patient group (18). However, in recent months, data on long COVID in pediatric patients has increasingly been published, giving new insights into the ways this phenomenon affects children. One Italian cross-sectional study showed that almost 53% of children had at least one persistent symptom 120 days or more after a diagnosis of SARS-CoV-2 infection (11). Similarly, report form Latvia showed that 51% of children had complaints 55 days after a diagnosis of SARS-CoV-2 infection, with 22% of patients noting 3 or more persistent simultaneous symptoms (12). Munblit et al. reported that almost 25% of children included in the study had at least one persistent symptom 256 days after being discharged from hospital, but 8.4% of children had complaints about multiple long-lasting symptoms. In addition, factors including being in an older age group (6–18 years) and having allergies were identified as the main risk factors for long COVID, but further in-depth research is needed to recognize these predisposing factors (10).

According to the latest studies, the most prevalent post-COVID symptoms in children are fatigue, headache, insomnia, sensory problems, cognitive sequelae, and post-viral catarrhal symptoms (i.e., cough, rhinorrhea, and sore throat) (10–14, 19, 20). The nature of long COVID is unpredictable, since persistent symptoms can be continuous or relapsing and remitting, making it difficult to establish a diagnose (21). Moreover, most of the reported persistent symptoms are not specific enough to separate long COVID from other medical conditions or, more importantly, pandemic-associated symptoms since the approach to mediating these symptoms is different.

Recently, Zimmerman et al., published the most comprehensive review article to date, discussing 14 studies investigating long COVID symptoms in children, and highlighting wide heterogeneity and methodological limitations, while also emphasizing the need to identify risk factors. This publication is believed potentially to be a turning point for development of further research in this field (16).

There is an urgent need to conduct further research into late sequelae of COVID-19, in particular by identifying the risk factors and mitigators (if any) for the development of long COVID, as the debate about the risks and benefits of COVID vaccinations among the pediatric age group intensifies. In addition, new global health policies related to early rehabilitation and reintegration possibilities need to be developed as the number of pediatric “long-haulers” increases (22).

## Materials and Methods

### Study Design and Participants

This was an ambidirectional cohort study focused on a pediatric population with the aim of identifying the long-term consequences of SARS-CoV-2 infection in children 1–6 months after acute COVID-19. It was conducted at the Children's Clinical University Hospital in Latvia, where a post-acute outpatient service for children after recovery from COVID-19 was established. The study population of COVID-19 patients who had been treated in outpatient settings as well as hospitals was enrolled between July 1, 2020, and April 30, 2021. Inclusion criteria were: (1) children aged 1 month to 18 years, (2) history of microbiologically confirmed (nasopharyngeal swab) SARS-CoV-2 infection or subsequent seroconversion, (3) acute phase of COVID-19 1–6 months before enrollment in the study. Exclusion criteria were: (1) patients with diagnosed Multisystem Inflammatory Syndrome in Children (MIS-C), (2) the family's unwillingness to participate in further follow-up, (3) no contact information or an inability to get in touch with the family (4) diagnosed co-infections, (5) COVID-19 onset more than 6 months previously. Patients or their parents/caregivers/legal guardians were interviewed “face-to face” according to a specially designed post-COVID-19 symptom assessment questionnaire, providing more accurate data collection and options for examining children during consultation (Supplementary Material 1). The protocol consisted of four main domains—physical and mental health, social, and psycho-emotional wellbeing. The questionnaire was based on previously published literature about long COVID in adults (5, 6, 8). During the follow-up, besides the post-acute symptoms, general demographical and epidemiological characteristics, as well as vaccination status, pre-existing chronic conditions, and clinical features during the acute phase of SARS-CoV-2 infection, were also collected.

In addition, between November 1, 2020, and April 30, 2021, patients with other non-SARS-CoV-2 community-acquired infections were also invited to participate in the study. This control group consisted of patients treated in outpatient settings as well as hospitalized children. Inclusion criteria were: (1) children aged 1 month to 18 years, (2) no history of COVID-19, (3) clinical and laboratory findings confirming other non-SARS-CoV-2 infections. Exclusion criteria for the comparison group were: (1) confirmed SARS-CoV-2 infection, (2) the family's unwillingness to participate in further follow-up, (3) no contact information or an inability to get in touch with the family. Eligible subjects in the comparison group or their parents/caregivers/legal guardians were asked the same interview questions as those in the cohort (Supplementary Material 2).

All interviews were carried out by our team of researchers involved in the study. The questionnaire was paper-based and written in Latvian. After interviews, data was transferred from questionnaires to MS Excel. Before the interview, caregivers had to provide written consent. The Ethics Committee of Riga Stradins University reviewed and approved the study protocol questionnaire and informed consent forms (approval No. 6-1/07/35).

### Definitions and Outcome Variables

To identify the long-term consequences of SARS-CoV-2 infection, we defined long COVID according to NICE's proposed definition as symptom persistence extending beyond 4 weeks from the onset of the first COVID-19 symptoms or a positive PCR test (1). Taking into consideration that patients in the study were enrolled in various time points (from 1 to 6 months after acute SARS-CoV-2 infection), all NICE's proposed clinical definitions were used throughout the manuscript according to timeframe.

Persistent symptoms were classified according to involved organ systems, including general sequelae, respiratory, cardiovascular, gastrointestinal, musculoskeletal, dermatological, neurological, and cognitive complaints.

### Procedure

Families whose children had recovered from SARS-CoV-2 infection were identified and invited to participate in the study in various direct and indirect ways. One of the first attempts to address the public directly was *via* a hospital communication campaign in the media (television, radio, and online news services). In this way, we invited parents to apply for their children to have an in-depth evaluation and long-term medical surveillance after SARS-CoV-2 infection. As well as the public, members of various professional medical associations also were asked to refer their pediatric COVID-19 patients for follow-up appointments in our hospital. In collaboration with the Disease Prevention and Control Center of Latvia, we also had direct contact with various general practitioners in whose practices confirmed pediatric COVID-19 outpatients had been treated, to offer our follow-up service. Finally, we co-operated with regional hospitals in various cities across Latvia (Valmiera, Kraslava, and Jekabpils), making it possible for our research team to organize regional consultations, so that we could help and support long COVID patients outside Riga.

The comparison group patients were included in the study in two ways. Firstly, children and their parents who had been treated in the Children's Clinical University Hospital due to their illness, were directly addressed and invited to participate. Secondly, direct contact with general practitioners in various regions of Latvia was established to invite pediatric patients to participate in the study.

### Statistical Analysis

Statistical analysis was performed using Statistical Package for the Social Sciences (SPSS) version 23.0 (IBM SPSS Corp.). Statistical significance was considered as *p* < 0.05. Standard methods [mean, standard deviation (SD), median for continuous variables, frequencies (expressed as percentages) for categorical variables] were used for descriptive statistics. Chi square or Fisher exact tests were used to check the significance of stratified frequencies of dependent variables between cases and controls. The Kolmogorov–Smirnov test was used to check whether the continuous variables met the criteria of normal distribution. The Mann–Whitney *U*-test was used to check the differences in continuous dependent variables between cases and controls. To check the impact of differences in age among cases and controls on the odds of post-acute and long COVID-19 symptoms, the adjustment was performed using binary and multinomial logistic regressions.

## Results

Overall, 239 COVID-19 patients were invited to participate in the study. Three declined to take part, and in all 236 COVID-19 patients with confirmed SARS-CoV-2 infection (positive PCR test or retrospective seroconversion) were enrolled. Of all the patients, 221 were symptomatic, but 15 children had no symptoms during the acute COVID-19 phase. Epidemiological data analysis showed that 80.1% (*N* = 189) of children had known contact with a SARS-CoV-2-positive person before acute symptom onset, but only 7.6% (*N* = 18) of patients had a history of recent travel. The median age of the study group was 10.0 years [Interquartile range (IQR), 5–14 years; range, 1 month−18 years], 55.5% (*N* = 131) of patients were boys. 86.9% (*N* = 205) of children were outpatients with mild disease, but 13.1 % (*N* = 31) had moderate or severe disease requiring hospitalization. The mean length of hospital stay was 3.8 (SD, 5.0) days. None of the hospitalized patients required admission to the Pediatric Intensive Care Unit (PICU). Fifty-two children (22%) had known pre-existing comorbidities. The most noted conditions were bronchial asthma, gastrointestinal disorders, psychiatric disorders, and epilepsy. No co-infections were diagnosed in the study group. Median follow-up time from acute symptom onset was 73.5 days (IQR; 43–110 days).

In all, 142 comparison group patients were also enrolled in the study. The median age was 2 years (IQR, 1–6 years; range 1 month−17 years), 53.5% (*N* = 76) were male. 57.7% (*N* = 82) were outpatients, but 42.3% (*N* = 60) of controls were treated in hospital with mean length of hospitalization being 3.9 (SD, 3.9) days. In this group, 16.9% (*N* = 24) of patients had known comorbidities. Bronchial asthma, allergies, and congenital heart disease were the most reported pre-existing conditions. The most often diagnosed non-SARS-CoV-2 community-acquired infections among the controls were the common cold (38.1%, *N* = 54), pharyngotonsillitis (21.8%, *N* = 31), gastrointestinal infections (19.7%, *N* = 28), urinary tract infections (8.5%, *N* = 12), pneumonia (3.5%, *N* = 5) and bacterial infections of unknown origin (3.5%, *N* = 5). Median follow-up time since acute symptom onset was 69 days (IQR, 58–84 days). There were significant statistical differences in age between the patients and controls (*p* < 0.001). Table 1 outlines the demographical data, comorbidities, and persistent symptom distribution across both study groups.

**Table 1:** Distribution of demographic features, comorbidities, and number of persistent symptoms in patients with COVID-19 and other non-SARS-CoV-2 infections.

	**COVID-19** ***N*** **=** **236**		**Non-SARS-CoV-2 viruses** ***N*** **=** **142**		***p*-values**	**Total**	
	***N***	**%**	***N***	**%**		***N***	**%**
**Median (interquartile range) age, years**	10.0 (14.0–5.0)		2.0 (6.0–1.0)		<0.001	6.0 (12.0–2.0)	
**Age group**							
1–11 months	18	7.6	13	9.2	<0.001	31	8.2
1–4 years	34	14.4	78	54.9		112	29.6
5–9 years	59	25.0	38	26.8		97	25.7
10–14 years	71	30.1	10	7.0		81	21.4
15–18 years	54	22.9	3	2.1		57	15.1
**Sex**							
Male	131	55.5	76	53.5	0.71	207	54.8
Female	105	44.5	66	46.5		171	45.2
**Vaccination**							
Seasonal influenza	26	11.9	24	17.6	0.13	50	14.1
BCG	233	98.7	–	–	–	233	98.7
MMR	208	95.4	–	–	–	208	95.4
**Comorbidities**							
Epilepsy	5	2.1	1	0.7	0.42	6	1.6
Gastrointestinal disorders	8	3.4	2	1.4	0.49	10	2.6
Bronchial asthma	17	7.2	10	7.0	0.95	27	7.1
Allergies	2	0.8	6	4.2	0.06	8	2.1
Congenital heart diseases	0	0	4	2.8	0.02	4	1.1
Atopic dermatitis	2	0.8	3	2.1	0.37	5	1.3
Rheumatic disorders	1	0.4	0	0	1.00	1	0.3
Cystic fibrosis	3	1.3	0	0	0.30	3	0.8
Urological disorders	1	0.4	1	0.7	1.00	2	0.5
Psychiatric disorders	6	2.5	0	0	0.09	6	1.6
Neurologic disorders	1	0.4	3	2.1	0.15	4	1.1
Congenital anomalies	3	1.3	0	0	0.30	3	0.8
**Post-acute follow-up characteristics**							
Days since symptoms onset, median (interquartile range)	73.5 (110.0–43.0)		69.0 (84.0–58.0)		0.36	70.0 (100.25–48.5)	
**Persistent symptoms**							
None	65	30.0	97	75.2	<0.001	162	46.8
1	35	16.1	16	12.4		51	14.7
2	19	8.8	10	7.8		29	8.4
≥3	98	45.2	6	4.7		104	30.1

**Figure 1:**
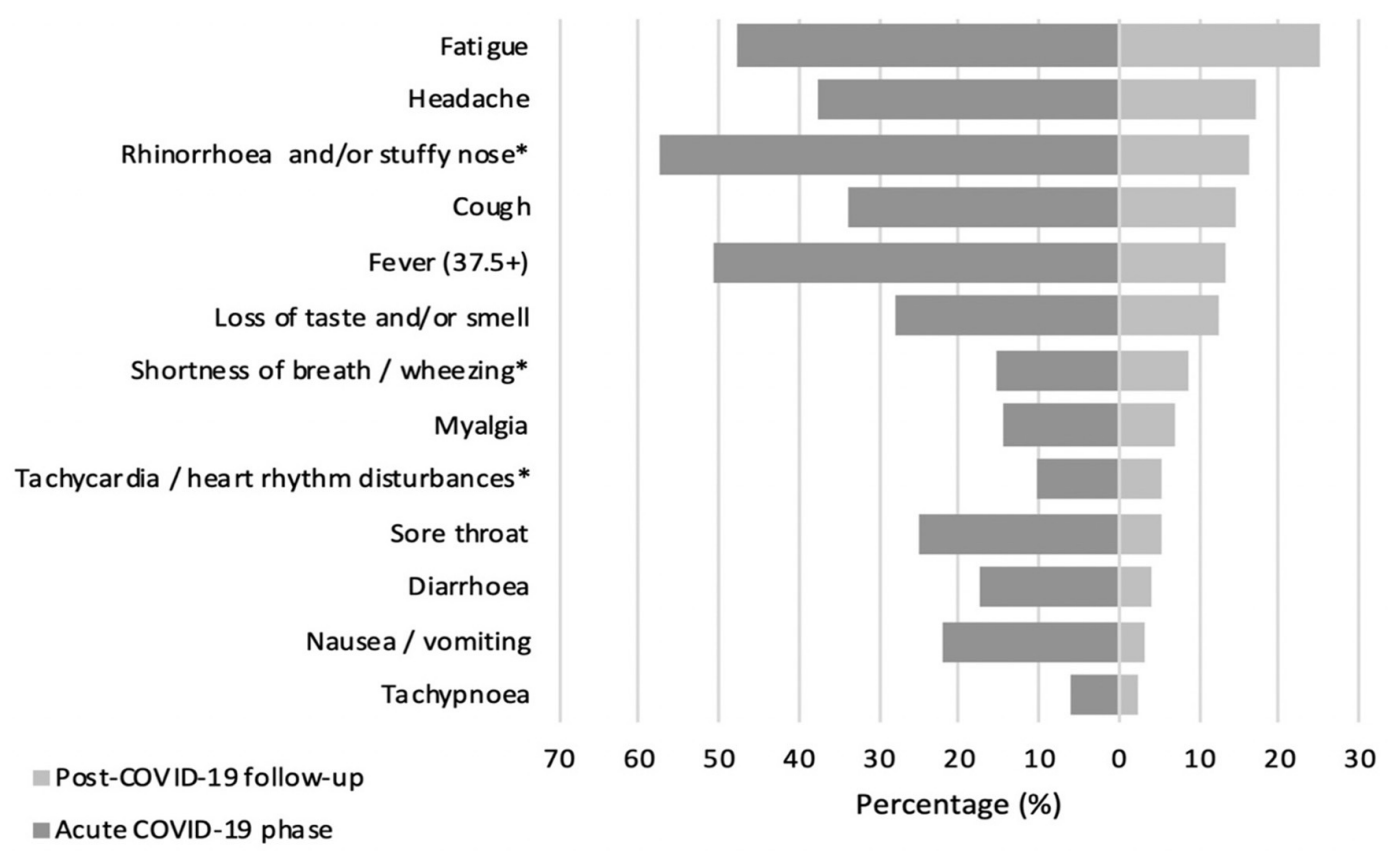
Prevalence (%) of COVID-19 associated symptoms in acute and post-acute phase. *Symptoms combined as they are similar.

At the time of the interview, only 30% (*N* = 65) of children had returned to their previous state of health, with no long COVID complaints. Most pediatric COVID-19 survivors (70%, *N* = 152) reported at least one persistent symptom, but more than half of the patients (54%, *N* = 117) noted multiple long-lasting symptoms, with an alarming tendency to experience three or more symptoms at the same time (45.2%, *N* = 98). Generally, the most reported complaints among COVID-19 patients concerned persistent fatigue (25.2%, *N* = 59), cognitive sequelae including irritability (24.3%, *N* = 57), mood changes (23.3%, *N* = 55), and impaired attention (16.9%, *N* = 40), as well as headaches (16.9%, *N* = 40), rhinorrhea (16.1%, *N* = 38), coughing (14.4%, *N* = 34), and disturbed taste and/or smell (12.3%, *N* = 29). Comparing the symptom spectrum in the acute infectious and long COVID phases, it was evident that catarrhal symptoms such as fever, rhinorrhea, cough, sore throat, and wheezing were more dominant in acute SARS-CoV-2 infection. On the other hand, symptoms as fatigue, headaches, and dysgeusia/anosmia were still frequently reported after acute illness. Figure 1 represents COVID-19 symptom distribution in the acute and post-acute phases.

The prevalence of general symptoms as well as cardiovascular (except for tachypnoea), musculoskeletal (except for stiffness), neurological, and cognitive sequelae was significantly higher in the COVID-19 patient group than among the comparison group (*p* < 0.05 for all). In addition, when comparing persistent symptom prevalence in 5–9-year-olds with ongoing symptomatic COVID-19 and other non-SARS-CoV-2 infections, we noticed that a loss of taste and/or smell, as well as cognitive sequelae (including mood changes, irritability, and anxiety/depression) were significantly higher in the COVID-19 patient group than among the comparison group (*p* < 0.05 for all). Logistic regression analysis showed that long-term persistent symptoms such as fever [adjusted Odds Ratio (ORa) = 4.0, 95% confidence interval (CI): 1.4–11.6, and *p* = 0.01], fatigue (ORa = 8.7, 95% CI: 2.5–29.9, and *p* = 0.001), rhinorrhea (ORa = 2.6, 95% CI: 1.3–5.4, and *p* = 0.008), loss of taste and/or smell (ORa = 11.2, 95% CI: 1.4–89.1, and *p* = 0.02), headaches (ORa = 12.9, 95% CI: 1.7–99.6, and *p* = 0.01), cognitive sequelae, as well as nocturnal sweating (ORa = 16.7, 95% CI: 2.1–130.4, and *p* = 0.007) were significantly associated with COVID-19 experience. Detailed information about persistent symptom prevalence and age-adjusted associations between COVID-19 status and persistent symptoms is presented in Table 2.

**Table 2:** Prevalence (%) of persistent symptoms in patients with COVID-19 and other non-SARS-CoV-2 infections and age-adjusted associations between COVID-19 status and persistent symptoms.

**Symptoms of long COVID 1–6 month after acute illness**	**COVID-19** ***N*** **=** **236**		**Non-SARS-CoV-2 infections** ***N*** **=** **142**		*p*-values^*^	ORa^**^	95% CI^***^	***p*-values**
	***N***	**%**	***N***	**%**				
**General sequelae**								
Fever	31	13.1	5	3.5	0.002	4.0	1.4–11.6	0.01
37.5–38°C	4	1.7	1	0.7	0.84	1.7	0.2–17.2	0.64
38.1–39.0°C	1	0.4	1	0.7		1.3	0.1–24.0	0.86
Fatigue	59	25.2	3	2.1	<0.001	8.7	2.5–29.9	0.001
**Respiratory sequelae**								
Cough	34	14.4	18	12.7	0.64	1.9	0.9–3.9	0.07
Sore throat	12	5.1	2	1.4	0.07	3.5	0.7–17.7	0.13
Wheezing	4	1.7	0	0	0.30	–	–	–
Rhinorrhea	38	16.1	16	11.3	0.19	2.6	1.3–5.4	0.008
Shortness of breath (at rest)	11	4.7	1	0.7	0.04	2.1	0.2–18.4	0.49
Shortness of breath (with physical activities)	17	7.2	0	0	0.001	–	–	–
**Otorhinolaryngological sequelae**								
Difficulty swallowing	2	0.8	0	0	0.53	–	–	–
Voice changes	6	2.5	0	0	0.09	–	–	–
Speech disturbances	3	1.3	0	0	0.30	–	–	–
Tinnitus	9	3.8	0	0	0.02	–	–	–
**Cardiovascular sequelae**								
Heart rhythm disturbances	12	5.1	0	0	0.005	–	–	–
Orthostatic intolerance	20	8.5	0	0	<0.001	–	–	–
Tachypnoea	6	2.5	0	0	0.09	–	–	–
**Gastrointestinal sequelae**								
Diarrhea	10	4.2	2	1.4	0.22	4.1	0.8–21.2	0.09
Vomiting/nausea	7	3.0	1	0.7	0.27	6.2	0.6–60.9	0.12
Loss of appetite	20	8.5	7	4.9	0.20	2.5	0.9–6.7	0.07
Body weight changes	20	8.5	3	2.1	0.01	2.3	0.6–8.7	0.22
**Musculoskeletal sequelae**								
Muscle pain	16	6.8	0	0	0.002	–	–	–
Muscle spasms	8	3.4	0	0	0.03	–	–	–
Joint pain	19	8.1	0	0	0.001	–	–	–
Stiffness	5	2.1	0	0	0.16	–	–	–
**Neurological sequelae**								
Loss of taste and/or smell	29	12.3	1	0.7	<0.001	11.2	1.4–89.1	0.02
Dizziness	21	8.9	0	0	<0.001	–	–	–
Headache	40	16.9	1	0.7	<0.001	12.9	1.7–99.6	0.01
Photophobia	12	5.1	0	0	0.005	–	–	–
**Cognitive sequelae**								
Difficulties to concentrate	40	16.9	1	0.7	<0.001	13.6	1.7–105.3	0.01
Impaired memory	24	10.2	1	0.7	<0.001	7.3	0.9–58.9	0.06
Impaired attention	40	16.9	1	0.7	<0.001	19.0	2.4–147.5	0.005
Mood changes	55	23.3	3	2.1	<0.001	16.0	4.6–55.2	<0.001
Irritability	57	24.3	3	2.1	<0.001	16.0	4.7–55.9	<0.001
Anxiety/depression	31	13.1	0	0	<0.001	–	–	–
**Dermatologic sequelae**								
Hair loss	6	2.5	0	0	0.09	–	–	–
**Other symptoms**								
Enlarged lymph nodes	6	2.5	0	0	0.08	–	–	–
Nocturnal sweating	23	9.7	1	0.7	<0.001	16.7	2.1–130.4	0.007
Menstrual disturbances	5	7.8	0	0	0.33	–	–	–

* *p-values for univariate analysis (Chi square or Fisher's exact test)*.

** *aOR, adjusted odds ratio, adjusted for age*.

*** *95% CI, 95% confidence interval*.

Comparing persistent symptom frequencies among hospitalized and non-hospitalized COVID-19 patients, in the former group children complained significantly more often of prolonged catarrhal symptoms, including coughing (32.3%, *N* = 10; vs. 11.7%, *N* = 24), rhinorrhea (41.9%, *N* = 13; vs. 12.2%, *N* = 25), as well as shortness of breath with physical activities (12.9%, *N* = 4; vs. 6.3%, *N* = 13). Interestingly, speech disturbances (6.5%, *N* = 2 vs. 0.5%, *N* = 1) and body weight changes (22.6%, *N* = 7; vs. 6.3%, *N* = 13) were also more prevalent in hospitalized patients. When sexes are compared, long-COVID symptoms tended to be more frequent among female patients. This tendency was prevalent across all long-term symptom subgroups, with the most significant dominance in cognitive and neurological sequelae (see Table 3).

**Table 3:** Prevalence (%) of persistent symptoms of COVID-19 according to the state of hospitalization, pre-existence of comorbidities, sex, age, and time since the onset of infection.

**Persistent symptoms of COVID-19, *N* (%)**	**All**	**Non-hospitalized**	**Hospitalized**	**Comorbidities**	**No comorbidities**	**Female**	**Male**	**Age < 1 years**	**Age 1–4 years**	**Age 5–9 years**	**Age 10–14 years**	**Age 15–18 years**	** <12 weeks**	**≥12 weeks**
	***N* = 236**	***N* = 205**	***N* = 31**	***N* = 52**	***N* = 184**	***N* = 105**	***N* = 131**	***N* = 18**	***N* = 34**	***N* = 59**	***N* = 71**	***N* = 54**	***N* = 131**	***N* = 105**
Fever	31 (13.1)	27 (13.2)	4 (12.9)	6 (11.5)	25 (13.6)	16 (15.2)	15 (11.5)	2 (11.1)	5 (14.7)	6 (10.2)	11 (15.5)	7 (13.0)	19 (14.5)	12 (11.4)
37.5°C−38°C	4 (1.7)	4 (2.0)	0	0	4 (2.0)	1 (1.0)	3 (2.3)	0	0	2 (3.4)	2 (2.8)	0	1 (0.8)	3 (2.9)
38.1°C−39.0°C	1 (0.4)	0	1 (3.2)	0	1 (0.5)	1 (1.0)	0	1 (5.6)	0	0	0	0	0	1 (1.0)
Fatigue	59 (25.2)	53 (26.0)	6 (20.0)	17 (33.3)	42 (23.0)	33 (31.7)	26 (20.0)^*^	2 (11.8)	5 (14.7)	10 (17.2)	22 (31.0)	20 (37.0)^*^	39 (30.0)	20 (19.2)
Cough	34 (14.4)	24 (11.7)	10 (32.3)^*^	10 (19.2)	24 (13.0)	18 (17.1)	16 (12.2)	2 (11.1)	10 (29.4)	9 (15.3)	10 (14.1)	3 (5.6)^*^	15 (11.5)	19 (18.1)
Sore throat	12 (5.1)	9 (4.4)	3 (9.7)	2 (3.8)	10 (5.4)	7 (6.7)	5 (3.8)	0	1 (2.9)	5 (8.5)	5 (7.0)	1 (1.9)	7 (5.3)	5 (4.8)
Wheezing	4 (1.7)	3 (1.5)	1 (3.2)	3 (5.8)	1 (0.5)^*^	3 (2.9)	1 (0.8)	0	0	1 (1.7)	3 (4.2)	0	2 (1.5)	2 (1.9)
Rhinorrhea	38 (16.1)	25 (12.2)	13 (41.9)^*^	11 (21.2)	27 (14.7)	24 (22.9)	14 (10.7)^*^	6 (33.3)	11 (32.4)	8 (13.6)	6 (8.5)	7 (13.0)^*^	21 (16.0)	17 (16.2)
Shortness of breath at rest	11 (4.7)	9 (4.4)	2 (6.5)	5 (9.6)	6 (3.3)	10 (9.5)	1 (0.8)^*^	0	0	1 (1.7)	7 (9.9)	3 (5.6)	7 (5.3)	4 (3.8)
Shortness of breath with physical activities	17 (7.2)	13 (6.3)	4 (12.9)	8 (15.4)	9 (4.9)^*^	13 (12.4)	4 (3.1)^*^	1 (5.6)	0	0	11 (15.5)	5 (9.3)^*^	9 (6.9)	8 (7.6)
Difficulty swallowing	2 (0.8)	2 (1.0)	0	0	2 (1.1)	2 (1.9)	0	0	0	1 (1.7)	1 (1.4)	0	0	2 (1.9)
Voice changes	6 (2.5)	5 (2.4)	1 (3.2)	1 (1.9)	5 (2.7)	5 (4.8)	1 (0.8)	1 (5.6)	1 (2.9)	2 (3.4)	2 (2.8)	0	2 (1.5)	4 (3.8)
Speech disturbances	3 (1.3)	1 (0.5)	2 (6.5)^*^	2 (3.8)	1 (0.5)	3 (2.9)	0	0	0	0	2 (2.8)	1 (1.9)	0	3 (2.9)
Tinnitus	9 (3.8)	8 (3.9)	1 (3.2)	4 (7.7)	5 (2.7)	6 (5.7)	3 (2.3)	0	0	0	6 (8.5)	3 (5.6)	5 (3.8)	4 (3.8)
Heart rhythm disturbances	12 (5.1)	10 (4.9)	2 (6.5)	5 (9.6)	7 (3.8)	10 (9.5)	2 (1.5)^*^	0	0	0	7 (9.9)	5 (9.3)^*^	10 (7.6)	2 (1.9)^*^
Orthostatic intolerance	20 (8.5)	18 (8.8)	2 (6.5)	7 (13.5)	13 (7.1)	14 (13.3)	6 (4.6)^*^	0	0	0	10 (14.1)	10 (18.5)^*^	13 (9.9)	7 (6.7)
Tachypnoea	6 (2.5)	4 (2.0)	2 (6.5)	2 (3.8)	4 (2.2)	5 (4.8)	1 (0.8)	0	1 (2.9)	0	4 (5.6)	1 (1.9)	4 (3.1)	2 (1.9)
Diarrhea	10 (4.2)	7 (3.4)	3 (9.7)	4 (7.7)	6 (3.3)	4 (3.8)	6 (4.6)	2 (11.1)	2 (5.9)	2 (3.4)	3 (3.4)	1 (1.9)	6 (4.6)	4 (3.8)
Vomiting/nausea	7 (3.0)	6 (2.9)	1 (3.2)	1 (1.9)	6 (3.3)	5 (4.8)	2 (1.5)	0	2 (5.9)	1 (1.7)	2 (2.8)	2 (3.7)	4 (3.1)	3 (2.9)
Loss of appetite	20 (8.5)	17 (8.3)	3 (9.7)	4 (7.7)	16 (8.7)	13 (12.4)	7 (5.3)	1 (5.6)	7 (20.6)	4 (6.8)	3 (4.2)	5 (9.3)	14 (10.7)	6 (5.7)
Body weight changes	20 (8.5)	13 (6.3)	7 (22.6)^*^	5 (9.6)	15 (8.2)	12 (11.4)	8 (6.1)	2 (11.1)	1 (2.9)	3 (5.1)	7 (9.9)	7 (13.0)	7 (5.3)	13 (12.4)
Muscle pain	16 (6.8)	14 (6.8)	2 (6.5)	7 (13.5)	9 (4.9)	11 (10.5)	5 (3.8)^*^	0	1 (2.9)	5 (8.5)	7 (9.9)	3 (5.6)	10 (7.6)	6 (5.7)
Muscle spasms	8 (3.4)	6 (2.9)	2 (6.5)	2 (3.8)	6 (3.3)	6 (5.7)	2 (1.5)	0	0	1 (1.7)	4 (5.6)	3 (5.6)	3 (2.3)	5 (4.8)
Joint pain	19 (8.1)	18 (8.8)	1 (3.2)	5 (9.6)	14 (7.6)	11 (10.5)	8 (6.1)	0	1 (2.9)	4 (6.8)	8 (11.3)	6 (11.1)	13 (9.9)	6 (5.7)
Stiffness	5 (2.1)	5 (2.4)	0	0	5 (2.7)	4 (3.8)	1 (0.8)	0	0	3 (5.1)	1 (1.4)	1 (1.9)	3 (2.3)	2 (1.9)
Loss of taste and/or smell	29 (12.3)	28 (13.7)	1 (3.2)	3 (5.8)	26 (14.1)	17 (16.2)	12 (9.2)	0	2 (5.9)	7 (11.9)	12 (16.9)	8 (14.8)	17 (13.0)	12 (11.4)
Dizziness	21 (8.9)	20 (9.8)	1 (3.2)	8 (15.7)	13 (7.1)	19 (18.1)	2 (1.5)^*^	0	1 (2.9)	0	9 (12.7)	11 (20.4)^*^	14 (10.8)	7 (6.7)
Headache	40 (16.9)	38 (18.5)	2 (6.5)	11 (21.2)	29 (15.8)	31 (29.5)	9 (6.9)^*^	0	1 (2.9)	9 (15.3)	16 (22.5)	14 (25.9)^*^	24 (18.3)	16 (15.2)
Photophobia	12 (5.1)	11 (5.4)	1 (3.2)	3 (5.8)	9 (4.9)	9 (8.6)	3 (2.3)^*^	0	1 (2.9)	1 (1.7)	3 (4.2)	7 (13.0)	7 (5.3)	5 (4.8)
Difficulties to concentrate	40 (16.9)	36 (17.6)	4 (12.9)	13 (25.0)	27 (14.7)	26 (24.8)	14 (10.7)^*^	0	2 (5.9)	7 (11.9)	16 (22.5)	15 (27.8)^*^	21 (16.0)	19 (18.1)
Impaired memory	24 (10.2)	21 (10.2)	3 (9.7)	10 (19.2)	14 (7.6)^*^	15 (14.3)	9 (6.9)	0	1 (2.9)	4 (6.8)	12 (16.9)	7 (13.0)	10 (7.6)	14 (13.3)
Impaired attention	40 (16.9)	34 (16.6)	6 (19.4)	14 (26.9)	26 (14.1)^*^	26 (24.8)	14 (10.7)^*^	0	4 (11.8)	8 (13.6)	15 (21.1)	13 (24.1)	20 (15.3)	20 (19.0)
Mood changes	55 (23.3)	47 (22.9)	8 (25.8)	18 (34.6)	37 (20.1)^*^	30 (28.6)	25 (19.1)	0	10 (29.4)	16 (27.1)	21 (29.6)	8 (14.8)^*^	27 (20.6)	28 (26.7)
Irritability	57 (24.3)	49 (24.0)	8 (25.8)	16 (31.4)	41 (22.3)	32 (30.5)	25 (19.2)^*^	1 (5.6)	10 (29.4)	16 (27.6)	20 (28.2)	10 (18.5)	28 (21.5)	29 (27.6)
Anxiety/depression	31 (13.1)	25 (12.2)	6 (19.4)	10 (19.2)	21 (11.4)	22 (21.0)	9 (6.9)^*^	0	1 (2.9)	7 (11.9)	14 (19.7)	9 (16.7)	14 (10.7)	17 (16.2)
Hair loss	6 (2.5)	4 (2.0)	2 (6.5)	3 (5.8)	3 (1.6)	6 (5.7)	0^*^	0	1 (2.9)	0	5 (7.0)	0	2 (1.5)	4 (3.8)
Enlarged lymph nodes	6 (2.5)	5 (2.4)	1 (3.2)	1 (1.9)	5 (2.7)	2 (1.9)	4 (3.1)	0	3 (8.8)	0	1 (1.4)	2 (3.7)	1 (0.8)	5 (4.8)
Nocturnal sweating	23 (9.7)	18 (8.8)	5 (16.1)	10 (19.2)	13 (7.1)^*^	12 (11.4)	11 (8.4)	4 (22.2)	2 (5.9)	8 (13.6)	7 (9.9)	2 (3.7)	12 (9.2)	11 (10.5)
Menstrual disturbances	5 (7.8)	4 (6.9)	1 (16.7)	2 (11.8)	3 (6.4)	5 (7.8)	–	–	–	0	3 (8.8)	2 (9.5)	3 (8.1)	2 (7.4)

* *p < 0.05*.

Comparing the prevalence of persistent symptoms across various age groups, among infants and toddlers, parents complained most frequently about persistent upper respiratory symptoms such as coughing (11.1%, *N* = 2 and 29.4%, *N* = 10) and rhinorrhea (33.3%, *N* = 6 and 32.4%, *N* = 11). Additionally, COVID-associated prolonged complaints such as diarrhea (11.1%, *N* = 2) and nocturnal sweating (22.2%, *N* = 4) were more often seen in infants than in any other age group. In older children, the most prevalent persistent symptoms were fatigue and cognitive disturbances, as well as neurological sequelae. Pre-schoolers and school-age children had post-COVID-associated irritability (27.6%, *N* = 16) and mood changes (27.1%, *N* = 16) among their most frequently reported symptoms. Interestingly, the prevalence of persistent fatigue and cognitive complaints increased considerably according to the study's age groups, with the highest rates seen among teenagers (14.7%, *N* = 5; 1–4-year-olds vs. 37.0%, *N* = 20; 15–18-year-olds; *p* = 0.04). Among children aged 10–14 years old, mood swings (29.6%, *N* = 21) were the most observed cognitive complaint. In contrast, in adolescents, cognitive disturbances including difficulty in concentrating (27.8%, *N* = 15) and an inability to focus their attention (24.1%, *N* = 13) were the most reported persistent symptoms; thus, many adolescents admitted that their performance at school had been seriously affected.

Taking into the consideration the timeframe of patient enrollment in the study, 105 pediatric COVID-19 patients were diagnosed with post-COVID-19 syndrome. Most frequently reported symptoms were irritability (27.6%, *N* = 29), mood changes (26.7%, *N* = 28) and fatigue (19.2%, *N* = 20). Interestingly, no statistical differences were seen among most reported persisting symptoms before and after 12-week cut-off point (with exception of heart rhythm disturbances, where statistically significant fall from 7.6% (*N* = 10) to 1.9% (*N* = 2) was seen **(**see Table 3).

## Discussion

Although research into long COVID among pediatric population is ongoing, and new information is published at tremendous speed all the time, our research has identified the significant number of children who experience long COVID. We found that 70% of children experienced long-term symptoms and, disturbingly, the majority of patients (53%) reported two or more concurrent symptoms. When attributing our pediatric population with symptom persistence (*N* = 152, 70%) to the total number of SARS-CoV-2 infected children in Latvia (as of July 27, 13,912 children had confirmed SARS-CoV-2 infection), it seems that only 1.09% of children infected with SARS-CoV-2 infection experienced persistent symptoms. We believe that this assumption could be representative, since our hospital is the only tertiary-level pediatric medical institution in Latvia in which a post-acute outpatient service for children recovering from COVID-19 could be established, and most children with long COVID are believed to have responded to our call to participate in the study. Long-term complaints were observed not only in hospitalized patients, but also in outpatients with asymptomatic or mild illness, emphasizing the variable nature of long COVID (23). Significantly, our study, similarly, to Australian data, refutes a serious misconception that if children get asymptomatic or mild SARS-CoV-2 infection, they aren't at risk of persistent post-acute symptoms (20). On the contrary, it is evident that long COVID can affect anyone, whatever their age. According to recent data published by the Office of National Statistics (in the United Kingdom), 12–15% of children may have symptoms persisting up to 5 weeks after an acute SARS-CoV-2 infection (24).

Crucially, the inclusion of the comparison group in the study allowed us to prove that symptom persistence is more evident with COVID-19 than any other non-SARS-CoV-2 infection. This conclusion resonates with all three outbreaks of highly pathogenic human coronaviruses (hCoVs) (25, 26). For example, Chinese researchers found that 1–3 months after SARS-CoV-1 infection, pediatric patients reported increased thinning of hair, decreased exercise tolerance, vague muscle weakness, emotional lability, and a transient decrease in attention span post-recovery (26). Up to this point, few viral infections had the potential to cause persistent symptoms in children. The most frequently discussed infection with late sequelae is Infectious Mononucleosis (IM), caused by the Epstein-Barr virus (EBV). For instance, research from the United States has revealed that 13% of adolescents met the criteria for Chronic Fatigue Syndrome (CFS) 6 months after acute EBV infection, but 7%, and 4% of children reported symptom persistence even as long as 12 and 24 months after acute infection, respectively (27).

Fatigue was the most frequently reported persisting symptom in our study—one in four children complained of this long-term effect. And many publications have proven that fatigue is the prevailing symptom across all age groups. Research from Russia showed that persistent fatigue was present in 10.6% of children, with a median follow-up time of 268 days (10). In contrast, a cross-sectional observational study from the Netherlands presented alarming data showing that up to 87% of pediatric COVID-19 survivors experienced persistent fatigue (28). Similar data has been reported from Sweden, showing fatigue as the most common symptom in hospitalized children, with a median follow-up time of 219 days (29). Among the adult population, a similar tendency has been observed, with 63% of hospitalized patients reporting persistent fatigue 6 months after acute COVID-19 (30).

Cognitive sequelae represented the second-most prevalent symptom group, with irritability and mood swings being the most frequent complaints. In another study, assessing 510 pediatric patients with long COVID, parents reported that ~55% of children had three or more mental health issues (31). In adults, the psychosocial distress and neuropsychiatric symptoms were more prominent, and recently there has been a growing awareness of these late-onset consequences of COVID-19. According to a recently published systematic review and meta-analysis by Badenoch et al. (32), sleep disturbance, fatigue, objectively measured cognitive impairment, anxiety, and Post-Traumatic Stress Disorder (PTSD) were the most common cognitive/neuropsychiatric symptoms in adults. In an Italian cohort of 402 adult COVID-19 survivors, 56% of patients showed at least one long-term psychiatric symptom (such as PTSD, depression, anxiety, insomnia, and obsessive-compulsive symptomatology) 1 month after hospitalization (33).

Many children in our study also had persistent respiratory symptoms, including rhinorrhea (16.1%) and coughing (14.4%), even though respiratory sequelae in general (dyspnea, cough, and chest pain) is more prevalent in adults (30, 34–38). A similar pattern was seen in another Italian study, in which 14.7% of the pediatric population had prolonged respiratory symptoms, including rhinorrhea (12.4%), chest tightness (6.2%), and persistent coughing (5.4%) (11). In addition, Australian researchers also found that the most common post-acute COVID-19 symptom in children was a mild post-viral cough (4%), lasting from 3 to 8 weeks from the time of symptom onset (20). We observed that upper respiratory-tract symptoms were most commonly reported among infants and preschoolers. This could be explained by the overwhelming volume of information available in the media about COVID-19, with the main emphasis on respiratory symptoms as the most common ones in children, thus shifting parents' focus on to the pandemic while neglecting other etiological agents of the common cold. Parents also emphasized that 13.1% of children had an elevated body temperature (as high as 37.5°C). As far as we are aware, fever (>37.5°C) is not widely associated with long COVID in children or adults. Tenforde et al. (34) have reported that in adult patients who complained about fever and chills on the day of testing for SARS-CoV-2, these symptoms were resolved in 97 and 96% of respondents within 14–21 days, respectively. Similarly, extensive reports on long-term fever in children are not available. Research from the Netherlands has shown that only two children from its study group had persistent fever (28).

Even though our study highlighted a wide spectrum of reported persistent COVID-19 symptoms with differences among the various age groups, the causation and correlation between late sequelae and SARS-CoV-2 infection remains unexplored, since biological underpinnings of persistent COVID-19 symptoms have not been studied sufficiently (36). Many of the reported long-COVID symptoms are non-specific, thus making it difficult to distinguish long COVID from pandemic-associated symptoms caused by lockdown measures (i.e., restricted socializing, school closures), which have been shown to have negative effects on the well-being and mental health of children and adolescents (16, 32, 39). In order to prove the correlation between SARS-CoV-2 infection and symptom persistence in children, it is crucial to carry out research with matched control groups and validated research tools, including objective functional testing and imaging, since self-reported symptoms may be difficult to validate (14, 16, 29).

Up to now, only five in pediatric population-based studies with included control groups have been published (13–15, 40, 41). The long-COVID prevalence rates are quite different, since only three of these studies found persistent symptoms to be more common in children with confirmed SARS-CoV-2 infection (13, 15, 40). The spectrum of the most commonly reported persistent symptoms was compatible with our study, since fatigue, headaches and prolonged respiratory symptoms were the most commonly reported complains. All these studies used self-or parent- reported persistent symptom questionnaires or online surveys, thus lacking the previously mentioned objective assessment. Furthermore, the follow-up time was variable in these studies, therefore they do not represent unified data on persistent symptom duration in children.

Recently, there have been some groundbreaking objective laboratory and imaging studies showing that long-term tissue damage, pathological inflammation, or dysimmune reactions may drive long COVID (23, 42–44). In addition, advanced imaging techniques can now identify possible underlying pathophysiological abnormalities of long COVID. For instance, Buonsenso et al. (45) have described using single-photon emission computed tomography with co-registered CT (SPECT/CT) to identify pulmonary circulation dysfunction with possible lung microvascular or endothelial damage in a 14-year-old girl with long COVID symptoms. Moreover, there have been novel indicators that a [^18^F]-FDG brain PET scan's visualized hypometabolic pattern could serve as a biomarker to help to identify severe or atypical forms of long COVID in children (46).

Undoubtedly, pediatric patients and their parents have contributed significantly to the recognition of long COVID in children with many parent-driven advocacy groups campaigning to draw awareness to this phenomenon, since resistance and doubts remain in the medical and scientific communities regarding the acceptance of long COVID in children. As the number of pediatric “long-haulers” increases, new global health policies are urgently needed to avoid misdiagnoses of long COVID and wrongful categorization of patients *via* an incorrect diagnosis (such as depression, anxiety, or fibromyalgia) (47).

As far as we are aware, our study is the first to compare symptom distribution across different age groups. Our study has highlighted that school-age children, and adolescents in particular, overwhelmingly complained of cognitive and neurological sequelae, but parents of infants and preschoolers more often reported respiratory symptoms. These differences in symptom distribution could be explained by the fact that older children could self-report and express their complaints to their parents and our research team at interview, whereas younger patients could not differentiate and express their subjective feelings. All data for younger children was obtained from their parents, whose judgment about their child's state of health may not always have been entirely accurate.

Our study has several strengths. Firstly, our follow-up was organized in the form of “face-to-face” visits, with the participation of parents and children themselves, to ensure better contact and communication with the families involved, and to provide more accurate data collection and options for examining children during consultations. Secondly, our study population included patients from all regions of Latvia, giving a better understanding of the overall picture across the country. Thirdly, our cohort consisted of both hospitalized and non-hospitalized patients, giving an accurate representation of the pediatric population. Fourthly, we enrolled a comparison group in our study, allowing us to compare a persistent symptom spectrum across SARS-CoV-2 and other non-SARS-CoV-2 infections, and to prove that long-term symptoms were more persistent with COVID-19.

Our study also faced limitations. Firstly, there was no clear case definition for long COVID in the pediatric population, thus making it difficult objectively to define patient inclusion criteria and follow-up times. Secondly, it was developed based on a non-validated questionnaire, and no objective parameters were used in patient evaluation. For reference, we began to design our study in early April 2020, when the first studies into the potential long-term consequences in adults began to emerge. Therefore, our questionnaire was designed according to those first reports. Thirdly, we started the patient enrolment at the peak of the first wave of pandemic. As we now look back, we acknowledge that some of the patients' reported symptoms could be psychosomatic or pandemic-associated, and not directly driven by COVID-19. Fourthly, there was a considerable age difference between both study groups due to pandemic- associated restrictions, which affected the homogeneity of our study population. For information, schools in Latvia were closed during the pandemic, and the only educational institutions open were day-care centers and kindergartens. Therefore, most of the children who turned to hospitals for help or outpatient care due to community-acquired non-SARS-CoV-2 infections were preschoolers, resulting in a younger comparison group than the COVID-19 patient group. Further control group's expansion and matching for age, sex, and testing time are needed.

Despite the study's limitations and pandemic-associated challenges, we believe that our contribution to long-COVID research is considerable, and our data may be used for further studies to continue to explore the nature, and consequences of long COVID in children. In addition, we are continuing further patient follow-up with repeated consultations at 12- and 24-months post-SARS-CoV-2 infection, thereby ensuring broader insight into persistent symptom distribution and prevalence among the pediatric population.

## Conclusions

This study highlights that long COVID represents a serious challenge to the pediatric population. Symptoms including persistent fatigue, cognitive sequelae, headaches, anosmia/dysgeusia, and respiratory sequelae were the most frequently reported complaints of long COVID-19, representing the wide range of symptoms affecting children. Comparison group of other non-SARS-CoV-2 infections allowed to prove that long-term symptoms are more prevalent among COVID-19 patients.

More research is needed to distinguish symptoms of long COVID from pandemic-associated complains. Each persistent symptom is important in terms of child well-being while recovering from COVID-19.

## Data Availability Statement

The raw data supporting the conclusions of this article will be made available by the authors, without undue reservation.

## Ethics Statement

The studies involving human participants were reviewed and approved by the Ethics Committee of Riga Stradins University (approval No. 6-1/07/35). Written informed consent to participate in this study was provided by the participants' legal guardian/next of kin.

## Author Contributions

IRo conceptualized and designed the study, designed the data collection instrument, collected data, reviewed the collected data, did the data analysis and interpretation, and drafted and revised the initial manuscript. LS conceptualized and designed the study, designed the data collection instrument, collected data, and contributed to the data analysis, interpretation, development of tables and figures, and drafted and revised the initial manuscript. AK-U consulted on the data collection instrument, did the statistical analysis of the collected data, and drafted and revised the initial manuscript. ZP, IRa, and LK collected data, reviewed the collected data, and drafted and revised the initial manuscript. JP conceptualized and designed the study, designed the data collection instruments, coordinated, and supervised the data collection, and critically reviewed the manuscript for important intellectual content. All authors approved the final manuscript as submitted and agreed to be accountable for all aspects of the work.

## Funding

This study was supported by the State Research Program for the Mitigation of Consequences of COVID-19, project Nr.VPP-COVID-2020/1-011: Impact of COVID-19 on the healthcare system and public health in Latvia; ways to prepare the health sector for future epidemics, funded by the Latvian Council of Science.

## Conflict of Interest

The authors declare that the research was conducted in the absence of any commercial or financial relationships that could be construed as a potential conflict of interest.

## Publisher's Note

All claims expressed in this article are solely those of the authors and do not necessarily represent those of their affiliated organizations, or those of the publisher, the editors and the reviewers. Any product that may be evaluated in this article, or claim that may be made by its manufacturer, is not guaranteed or endorsed by the publisher.

## References

[1] National Institute for Health and Care Excellence. COVID-19 Rapid Guideline: Managing the Long-Term Effects of COVID-19. (2020). Available online at: www.nice.org.uk/guidance/ng188 (accessed June 15, 2021). 33555768

[2] Amenta EM Spallone A Rodriguez-Barradas MC El Sahly HM Atmar RL Kulkarni PA. Postacute COVID-19: an overview and approach to classification. Open Forum Infect Dis. (2020) 7:ofaa509. 10.1093/ofid/ofaa509 33403218 PMC7665635

[3] Fernández-de-Las-Peñas C Palacios-Ceña D Gómez-Mayordomo V Cuadrado ML Florencio LL. Defining post-COVID symptoms (post-acute COVID, long COVID, persistent post-COVID): an integrative classification. Int J Environ Res Public Health. (2021) 18:2621. 10.3390/ijerph18052621 33807869 PMC7967389

[4] Greenhalgh T Knight M A'Court C Buxton M Husain L. Management of post-acute covid-19 in primary care. BMJ. (2020) 370:m3026. 10.1136/bmj.m3026 32784198

[5] Del Rio C Collins LF Malani P. Long-term Health Consequences of COVID-19. JAMA. (2020) 324:1723–4. 10.1001/jama.2020.19719 33031513 PMC8019677

[6] Moreno-Pérez O Merino E Leon-Ramirez JM Andres M Ramos JM Arenas-Jiménez J. Post-acute COVID-19 syndrome. Incidence and risk factors: a Mediterranean cohort study. J Infect. (2021) 82:378–83. 10.1016/j.jinf.2021.01.004 33450302 PMC7802523

[7] Carfì A Bernabei R Landi F; Gemelli Against COVID-19 Post-Acute Care Study Group. Persistent symptoms in patients after acute COVID-19. JAMA. (2020) 324:603–5. 10.1001/jama.2020.12603 32644129 PMC7349096

[8] Kings College London, COVID Symptom Study. How Long Does COVID-19 Last? (2020). Available online at: https://covid19.joinzoe.com/post/covid-long-term?fbclid=IwAR1RxIcmmdL-EFjh_aI- (accessed June 17, 2021).

[9] Xiong Q Xu M Li J Liu Y Zhang J Xu Y. Clinical sequelae of COVID-19 survivors in Wuhan, China: a single-center longitudinal study. Clin Microbiol Infect. (2021) 27:89–95. 10.1016/j.cmi.2020.09.023 32979574 PMC7510771

[10] Osmanov IM Spiridonova E Bobkova P Gamirova A Shikhaleva A Andreeva M. Risk factors for long covid in previously hospitalised children using the ISARIC Global follow-up protocol: a prospective cohort study. Eur Respir J. (2021) 58:2101341. 10.1101/2021.04.26.21256110 34210789 PMC8576804

[11] Buonsenso D Munblit D De Rose C Sinatti D Ricchiuto A Carfi A. Preliminary evidence on long COVID in children. Acta Paediatr. (2021) 110:2208–11. 10.1111/apa.15870 33835507 PMC8251440

[12] Smane L Stars I Pucuka Z Roge I Pavare J. Persistent clinical features in paediatric patients after SARS-CoV-2 virological recovery: a retrospective population-based cohort study from a single centre in Latvia. BMJ Paediatr Open. (2020) 4:e000905. 10.1136/bmjpo-2020-000905 34192185 PMC7778738

[13] Molteni E Sudre CH Canas LS Bhopal SS Hughes RC Antonelli M. Illness duration and symptom profile in symptomatic UK school-aged children tested for SARS-CoV-2. Lancet Child Adolesc Health. (2021) 5:708–18. 10.1016/S2352-4642(21)00198-X 34358472 PMC8443448

[14] Radtke T Ulyte A Puhan MA Kriemler S. Long-term symptoms after SARS-CoV-2 infection in children and adolescents. JAMA. (2021) 326:869–71. 10.1001/jama.2021.11880 34264266 PMC8283661

[15] Stephenson T Shafran R De Stavola B Rojas N Aiano F Amin-Chowdhury Z. Long COVID and the mental and physical health of children and young people: national matched cohort study protocol (the CLoCk study). BMJ Open. (2021) 11:e052838. 10.1136/bmjopen-2021-052838 34446502 PMC8392739

[16] Zimmermann P Pittet LF Curtis N. How common is long COVID in children and adolescents? Pediatr Infect Dis J. (2021). 10.1097/INF.0000000000003328. [Epub ahead of print]. PMC8575095 34870392

[17] Centers for Disease Control and Prevention. Evaluating and Caring for Patients with Post-COVID Conditions: Interim Guidance. (2021). Available online at: https://www.cdc.gov/coronavirus/2019-ncov/hcp/clinical-care/post-covid-index.html (accessed June 17, 2021).

[18] Hoang A Chorath K Moreira A Evans M Burmeister-Morton F Burmeister F. COVID-19 in 7780 pediatric patients: a systematic review. EClinicalMedicine. (2020) 24:100433. 10.1016/j.eclinm.2020.100433 32766542 PMC7318942

[19] Ludvigsson JF. Case report and systematic review suggest that children may experience similar long-term effects to adults after clinical COVID-19. Acta Paediatr. (2021) 110:914–21. 10.1111/apa.15673 33205450 PMC7753397

[20] Say D Crawford N McNab S Wurzel D Steer A Tosif S. Post-acute COVID-19 outcomes in children with mild and asymptomatic disease. Lancet Child Adolesc Health. (2021) 5:e22–e3. 10.1016/S2352-4642(21)00124-3 33891880 PMC8057863

[21] Nabavi N. Long covid: how to define it and how to manage it. BMJ. (2020) 370:m3489. 10.1136/bmj.m3489 32895219

[22] Hageman JR. Long COVID-19 or Post-acute sequelae of SARS-CoV-2 infection in children, adolescents, and young adults. Pediatr Ann. (2021) 50:e232–e3. 10.3928/19382359-20210519-02 34115558

[23] Salamanna F Veronesi F Martini L Landini MP Fini M. Post-COVID-19 syndrome: the persistent symptoms at the post-viral stage of the disease. A Systematic Review of the Current Data. Front Med. (2021) 8:653516. 10.3389/fmed.2021.653516 34017846 PMC8129035

[24] Office for National Statistics. Updated Estimates of the Prevalence of Long COVID Symptoms. (2021). Available online at: https://www.ons.gov.uk/peoplepopulationandcommunity/healthandsocialcare/healthandlifeexpectancies/adhocs/12788updatedestimatesoftheprevalenceoflongcovidsymptoms (accessed June 20, 2021).

[25] Yu CC Li AM So RC McManus A Ng PC Chu W. Longer term follow up of aerobic capacity in children affected by severe acute respiratory syndrome (SARS). Thorax. (2006) 61:240–6. 10.1136/thx.2005.046854 16449271 PMC2080724

[26] Leung CW Kwan YW Ko PW Chiu SS Loung PY Fong NC. Severe acute respiratory syndrome among children. Pediatrics. (2004) 113:e535–43. 10.1542/peds.113.6.e535 15173534

[27] Katz BZ Shiraishi Y Mears CJ Binns HJ Taylor R. Chronic fatigue syndrome after infectious mononucleosis in adolescents. Pediatrics. (2009) 124:189–93. 10.1542/peds.2008-1879 19564299 PMC2756827

[28] Brackel CLH Lap CR Buddingh EP van Houten MA van der Sande LJTM Langereis EJ. Pediatric long-COVID: an overlooked phenomenon? Pediatr Pulmonol. (2021) 56:2495–502. 10.1002/ppul.25521 34102037 PMC8242715

[29] Sterky E Olsson-Åkefeldt S Hertting O Herlenius E Alfven T Ryd Rinder M. Persistent symptoms in Swedish children after hospitalisation due to COVID-19. Acta Paediatr. (2021) 110:2578–80. 10.1111/apa.15999 34157167 PMC8444740

[30] Huang C Huang L Wang Y Li X Ren L Gu X. 6-month consequences of COVID-19 in patients discharged from hospital: a cohort study. Lancet. (2021) 397:220–32. 10.1016/S0140-6736(20)32656-8 33428867 PMC7833295

[31] Badenoch JB Rengasamy E Watson CJ Jansen K Chakraborty S Sundaram RD. Persistent neuropsychiatric symptoms after COVID-19: a systematic review an, meta-analysis. medRxiv [Preprint]. (2021). 10.1101/2021.04.30.21256413 PMC8833580 35169700

[32] Buonsenso D Espuny Pujol F Munblit D Mcfarland S Simpson F. Clinical characteristics, activity levels and mental health problems in children with long COVID: a survey of 510 children. Preprints. (2021). 10.20944/preprints202103.0271.v1 PMC9248023 35360923

[33] Mazza MG De Lorenzo R Conte C Poletti S Vai B Bollettini I. Anxiety and depression in COVID-19 survivors: role of inflammatory and clinical predictors. Brain Behav Immun. (2020) 89:594–600. 10.1016/j.bbi.2020.07.037 32738287 PMC7390748

[34] Tenforde MW Kim SS Lindsell CJ Billig Rose E Shapiro NI Files DC. Symptom duration and risk factors for delayed return to usual health among outpatients with COVID-19 in a Multistate Health Care Systems Network - United States, March-June 2020. MMWR Morb Mortal Wkly Rep. (2020) 69:993–8. 10.15585/mmwr.mm6930e1 32730238 PMC7392393

[35] Nalbandian A Sehgal K Gupta A Madhavan MV McGroder C Stevens JS. post-acute COVID-19 syndrome. Nat Med. (2021) 27:601–15. 10.1038/s41591-021-01283-z 33753937 PMC8893149

[36] Halpin SJ McIvor C Whyatt G Adams A Harvey O McLean L. Postdischarge symptoms and rehabilitation needs in survivors of COVID-19 infection: a cross-sectional evaluation. J Med Virol. (2021) 93:1013–22. 10.1002/jmv.26368 32729939

[37] Raveendran AV Jayadevan R Sashidharan S. Long COVID: an overview. Diabetes Metab Syndr. (2021) 15:869–75. 10.1016/j.dsx.2021.04.007 33892403 PMC8056514

[38] Carvalho-Schneider C Laurent E Lemaignen A Beaufils E Bourbao-Tournois C Laribi S. Follow-up of adults with noncritical COVID-19 two months after symptom onset. Clin Microbiol Infect. (2021) 27:258–63. 10.1016/j.cmi.2020.09.052 33031948 PMC7534895

[39] de Figueiredo CS Sandre PC Portugal LCL Mázala-de-Oliveira T da Silva Chagas L Raony Í. COVID-19 pandemic impact on children and adolescents' mental health: biological, environmental, and social factors. Prog Neuropsychopharmacol Biol Psychiatry. (2021) 106:110171. 10.1016/j.pnpbp.2020.110171 33186638 PMC7657035

[40] Miller FM Nguyen V Navaratnam AMD Shrotri M Kovar J Hayward AC. Prevalence of persistent symptoms in children during the COVID-19 pandemic: evidence from a household cohort study in England and Wales. MedRxiv [Preprints]. (2021). 10.1101/2021.05.28.21257602 PMC9645448 36375098

[41] Blankenburg J Wekenborg MK Reichert J Kirsten C Kahre E Haag L. Mental health of Adolescents in the pandemic: long-COVID19 or long-pandemic syndrome? MedRxiv [Preprints]. (2021) 10.1101/2021.05.11.21257037

[42] Proal AD VanElzakker MB. Long COVID or Post-acute sequelae of COVID-19 (PASC): an overview of biological factors that may contribute to persistent symptoms. Front Microbiol. (2021) 12:698169. 10.3389/fmicb.2021.698169 34248921 PMC8260991

[43] Yong SJ. Long COVID or post-COVID-19 syndrome: putative pathophysiology, risk factors, and treatments. Infect Dis (Lond). (2021) 53:737–54. 10.1080/23744235.2021.1924397 34024217 PMC8146298

[44] Guilmot A Maldonado Slootjes S Sellimi A Bronchain M Hanseeuw B Belkhir L. Immune-mediated neurological syndromes in SARS-CoV-2-infected patients. J Neurol. (2021) 268:751–7. 10.1007/s00415-020-10108-x 32734353 PMC7391231

[45] Buonsenso D Di Giuda D Sigfrid L Pizzuto DA Di Sante G De Rose C. Evidence of lung perfusion defects and ongoing inflammation in an adolescent with post-acute sequelae of SARS-CoV-2 infection. Lancet Child Adolesc Health. (2021) 5:677–80. 10.1016/S2352-4642(21)00196-6 34339624 PMC8324416

[46] Morand A Campion JY Lepine A Bosdure E Luciani L Cammilleri S. Similar patterns of [18F]-FDG brain PET hypometabolism in paediatric and adult patients with long COVID: a paediatric case series. Eur J Nucl Med Mol Imaging. (2021) 48:1–8. 10.21203/rs.3.rs-722537/v1 PMC8376118 34414470

[47] Buonsenso D Fusco C De Rose C Valentini P Vergari J. Long COVID in children: partnerships between families and paediatricians are a priority for better care. J Paediatr Child Health. (2021). 10.1111/jpc.15600. [Epub ahead of print]. 34060675 PMC8242393

