# Supplementary material for: Comparison of Persistent Symptoms After COVID-19 and Other Non-SARS-CoV-2 Infections in Children
Source: Front Pediatr. 2021 Oct 29;9:752385. doi: 10.3389/fped.2021.752385 (PMC8586002; doi:10.3389/fped.2021.752385)
Supplement: Supplementary file 1 [file Data_Sheet_1.PDF]

Post-non-SARS-CoV-2 infection symptom assessment questionnaire

Part I: General information, the acute disease phase				
**1.**	**Family doctor, telephone number**			
**2.**	**Date of consultation**			
**3.**	**Patient number**			
**4.**	**Age**	Months		Years
**5.**	**Sex**	Female		Male
**6.**	**Day of first symptoms with current infection?**			
**7.**	**Does the child have any comorbidities? (Rheumatological, cardiological, respiratory, immunodeficiency, diabetes etc.)**	Yes (*please specify*)		
		No		
**8.**	**Is the child taking medication to treat any chronic diseases?**	Yes (*please specify*)		
		No		
**9.**	**Has vaccination been completed following the Latvian immunisation calendar?**	Full immunisation		
		Please clarify: did the child receive the BCG vaccination?		
		Please clarify: Did the child receive the MMR vaccination?		
		Partial immunisation		
		No vaccination		
**10.**	**Has the child received immunisation against influenza for this flu season?**	Yes		
		No		
	**Interview** with Mother/ Father/Other<br><br>**Mother** – Basic education/Secondary education/ Higher education/Unfinished higher education/ Vocational education.<br><br>**Father** – Basic education/Secondary education/ Higher education/Unfinished higher education/ Vocational education.	**Mother** – Married /Living together / Divorced /Other family/Separated<br><br>**Father** – Married /Living together / Divorced/ Other family / Separated		

**11.**	11.1 Most common symptoms	Fever (temperature measured axillary):<br>○ 37,5 – 38,0 °C<br>○ 38,1 – 39,0 °C<br>○ 39,0 °C	
		Cough	
		Sore throat	
		Sneezing	
		Shortness of breath	
		Wheezing	
		Muscle pain	

Post-non-SARS-CoV-2 infection symptom assessment questionnaire

	11.2 Other symptoms	Fatigue	
		Headache	
		Rhinorrhoea	
		Stuffy nose	
		Loss of taste/smell	
		Tachypnoea	
		Tachycardia	
		Oxygen saturation <92%	
		Abdominal pain	
		Diarrhoea	
		Vomiting	
		Nausea	
		Other	

Investigation

**12.**	12.1	Have not been performed		
	12.2	Have been done (please, mark which):		
	12.2.1	COVID-19 PCR test	Done	Negative
	12.2.2	Full blood count	Done	Any abnormalities? Please specify.
	12.2.3	CRP	Done	CRP value (mg/dL):
	12.2.4	Rapid Strep A test	Done	Positive
				Negative
	12.2.5	Rapid Influenza antigen test	Done	Positive
				Negative
	12.2.6	Rapid RSV antigen test	Done	Positive
				Negative
	12.2.7	Chest X-ray	Done	Any abnormalities? Please specify.
	12.2.8	Others (please, specify)		

Treatment

**13.**	**Type of treatment:**	How many days did the treatment last?
	Peroral rehydration	
	Antipyretic treatment	
	Inhalations	
	Antibacterial therapy	
	Intravenous rehydration	
	Other	

Part II: Non-SARS-CoV-2 patient follow-up

**14. Did the child have any other acute infections following this episode?**		**1–3-month follow-up**	**6-month follow-up**	**12-month follow-up**	**24-month follow-up**
**Date of consultation**					
Yes	Please specify				

Post-non-SARS-CoV-2 infection symptom assessment questionnaire

No					
14.1 Received treatment during illness					
	- Antibacterial therapy				
	- P/o rehydration				
	- Antipyretic therapy				
	- Inhalations				
	- Other				
15. What kind of medications/vitamins does the child receive daily?					
16. Has the child received a vaccine against influenza in the autumn/winter season 2020/2021?					
17. Does the child have any persistent symptoms following acute infection episode?					
**Catarrhal symptoms**					
18.1 Elevated body temperature					
	- How many degrees (C^{o})?				
	- How often was the T measured?				
18.2 Cough					
18.3 Sore throat					
18.4 Wheezing					
18.5 Rhinorrhoea					
18.6 Shortness of breath					
	- In a resting state				
	- While doing physical activities				
18.7 Enlarged lymph nodes					
**Otorhinolaryngological sequelae**					
19.1 Difficulty swallowing					
19.2 Voice changes					

19.3 Speech disturbances				
19.4 Tinnitus				
**Cardiovascular sequelae**				
20.1 Heart rhythm disturbances (including tachycardia)				
20.2 Orthostatic intolerance				
20.3 Tachypnoea				
**Gastrointestinal sequelae**				
21.1 Diarrhoea				
21.2 Vomiting/nausea				
21.3 Loss of appetite				
21.4 Body weight changes				
**Musculoskeletal sequelae**				
22.1 Muscle pain				
22.2 Muscle spasms				
22.3 Joint pain				
22.4 Stiffness				
**Exercise intolerance**				
23. Exercise Intolerance				
- Which physical activities can't be done?				
- How long can physical activity be tolerated (minutes)?				
- Does the physical activity provoke increased fatigue/sleepiness?				
- Do the symptoms disappear when lying down?				
- Is it hard to walk for longer than 15 minutes?				
- Is it hard to get up more than two flights of stairs?				
- Is there any physical activity that is more difficult than previously?				

Post-non-SARS-CoV-2 infection symptom assessment questionnaire

Day-to-day activities and sleep disorders					
24. Increased fatigue					
	- Are there any provoking factors which amplify fatigue?				
	- How many hours a night does the child sleep?				
	- Is the child tired in the morning, despite a good night's sleep?				
	- Are there any day-to-day activities (dressing, washing etc.) that are more difficult now than they were before?				
25. Night sweating					
**Neurological sequelae**					
26.1 Loss of taste and/or smell					
26.2 Dizziness					
26.3 Headache					
26.4 Photophobia					
**Cognitive sequelae**					
27.1 Difficulties in concentrating					
27.2 Impaired memory					
27.3 Impaired attention					
27.4 Mood changes					
27.5 Irritability					
27.6 Anxiety/depression					
27.7 Other					

Other Symptoms				
28.1 Hair loss				
28.2 Menstrual disturbances				
28.3 Others				

Post-non-SARS-CoV-2 infection symptom assessment questionnaire

29. How many days has the child not attended school/kindergarten after recovering from the infection because of the above symptoms?					
30. How many times has the child visited the family doctor after recovering from infection?					
31. Has the child visited a specialist after recovering from infection?					
	What kind of specialist? Why?				
**Examination**					
32. What studies have been performed after recovery from acute infection?					
32.1 Laboratory exams					
	- F.B.C.				
	- CRP/Il-6				
	- Urine analysis				
	- Creatinine/urea				
	- ALAT/ASAT				
	- Antibodies against SARS-CoV-2				
	- Others				
32.2 Imaging exams					
	- X-ray				
	- USG				
	- Others				
