# Supplementary material for: Comparison of Persistent Symptoms After COVID-19 and Other Non-SARS-CoV-2 Infections in Children
Source: Front Pediatr. 2021 Oct 29;9:752385. doi: 10.3389/fped.2021.752385 (PMC8586002; doi:10.3389/fped.2021.752385)
Supplement: Supplementary file 2 [file Data_Sheet_2.PDF]

Post-COVID-19 symptom assessment questionnaire

Part I: General information, the acute disease phase				
1.	Family doctor, telephone number			
2.	Date of consultation			
3.	Patient number			
4.	Age	Months		Years
5.	Sex	Female		Male
6.	Day of first symptoms with COVID-19?			
7.	Does the child have any comorbidities? (Rheumatological, cardiological, respiratory, immunodeficiency, diabetes etc.)	Yes (*please specify*)		
		No		
8.	Is the child taking medication to treat any chronic diseases?	Yes (*please specify*)		
		No		
9.	Has vaccination been completed following the Latvian immunisation calendar?	Full immunisation		
		Please clarify: did the child receive the BCG vaccination?		
		Please clarify: Did the child receive the MMR vaccination?		
		Partial immunisation		
		No vaccination		
10.	Has the child received immunisation against influenza for this flu season?	Yes		
		No		
11.	Date of COVID-19 test (date of positive response)			
12.	Was repeated testing carried out (date/result)?			
	**Interview** with Mother/ Father/Other<br><br>**Mother** – Basic education/Secondary education/ Higher education/Unfinished higher education/ Vocational education.<br><br>**Father** – Basic education/Secondary education/ Higher education/Unfinished higher education/ Vocational education.	**Mother –** Married /Living together / Divorced /Other family/Separated<br><br>**Father –** Married /Living together / Divorced/ Other family / Separated		

13.	13.1 Most common symptoms	Fever (temperature measured axillary):<br>○ 37,5 – 38,0 °C<br>○ 38,1 – 39,0 °C<br>○ 39,0 °C	
		Cough	
		Sore throat	
		Sneezing	
		Shortness of breath	
		Wheezing	

Post-COVID-19 symptom assessment questionnaire

	13.2 Other symptoms	Muscle pain	
		Fatigue	
		Headache	
		Rhinorrhoea	
		Stuffy nose	
		Loss of taste/smell	
		Tachypnoea	
		Tachycardia	
		Oxygen saturation <92%	
		Abdominal pain	
		Diarrhoea	
		Vomiting	
		Nausea	
		Other	

Epidemiological History			
**14.**	14.1	Data about travelling in the last 14 days.	Yes
			No
	14.2	Has been in contact with COVID-19 virus RNA positive patient in the last 14 days.	Yes
			No
	14.3	Has been in contact with a person who has been tested for COVID-19, with an unknown test result.	Yes
			No
	14.4	Has been in contact with a person with fever and/or respiratory symptoms (children may also have gastrointestinal symptoms) and contact with epidemiological communities where there have been laboratory-confirmed cases of COVID-19 infection in the last 14 days.	Yes
			No
	14.5	Has been in contact with group cases (more than 2 cases of fever and/or respiratory symptoms via, for example, families, offices, school classes and other small communities in the last 14 days).	Yes
			No
	14.6	Patient is from a care centre with laboratory-conformed cases of COVID-19 infection over the past 14 days.	Yes
			No

Laboratory/radiological exams performed at outpatient stage			
**15.**	Full blood count		Yes
			No
	Were there any abnormalities?		
	C-reactive protein, mg/L		Yes
			No
	IL-6, pg/mL		Yes
			No
	ALAT, ASAT, U/L, Urea, mmol/L, Creatinine, umol/L		Yes
			No
	CK-MB, ng/mL, LDH, U/L		Yes
			No
	RTG thoracis or other radiological exams, or functional examinations		Yes
			No

Post-COVID-19 symptom assessment questionnaire

	D-dimer, mg/L FEU, ESR, mm/h, Creatininkinasis, U/L	Yes
		No
	Ferritin, ng/mL	Yes
		No
	Urine analysis	Yes
		No
	Other examinations	Yes
		No

**Treatment received at outpatient stage**	
**16. Type of treatment**	How many days did the treatment last?
P/o rehydration	
Antipyretic therapy	
Inhalations	
Antibacterial therapy	
Other	

**Part II: COVID-19 patient follow-up**					
**17. Did the child have any other acute infections following COVID-19?**		**1–3-month follow-up**	**6-month follow-up**	**12-month follow-up**	**24-month follow-up**
**Date of consultation**					
Yes	Please specify				
No					
17.1 Received treatment during illness					
	- Antibacterial therapy				
	- P/o rehydration				
	- Antipyretic therapy				
	- Inhalations				
	- Other				
18. What kind of medication/vitamins does the child receive daily?					
19. Has the child received a vaccine against influenza in the autumn/winter season 2020/2021; 2021/2022?					

Post-COVID-19 symptom assessment questionnaire

20. Does the child have any persistent symptoms in the wake of COVID-19?					
**Catarrhal symptoms**					
20.1 Elevated body temperature (axillary)					
	- How many degrees (C°)?				
	- How often was the T measured?				
20.2 Cough					
20.3 Sore throat					
20.4 Wheezing					
20.5 Rhinorrhoea					
20.6 Shortness of breath					
	- In a resting state				
	- While doing physical activities				
20.7 Enlarged lymph nodes					
**Otorhinolaryngological sequelae**					
20.8 Difficulty swallowing					
20.9 Voice changes					
20.10 Speech disturbances					
20.11 Tinnitus					
**Cardiovascular sequelae**					
20.12 Heart rhythm disturbances (including tachycardia)					
20.13 Orthostatic intolerance					
20.14 Tachypnoea					
**Gastrointestinal sequelae**					
20.15 Diarrhoea					
20.16 Vomiting/nausea					

Post-COVID-19 symptom assessment questionnaire

20.17 Loss of appetite					
20.18 Body weight changes					
**Musculoskeletal sequelae**					
20.19 Muscle pain					
20.20 Muscle spasms					
20.21 Joint pain					
20.22 Stiffness					
**Exercise intolerance**					
20.23 Exercise Intolerance					
	- Which physical activities can't be done?				
	- How long can physical activity be tolerated (minutes)?				
	- Does the physical activity provoke increased fatigue/sleepiness?				
	- Do the symptoms disappear when lying down?				
	- Is it hard to walk for longer than 15 minutes?				
	- Is it hard to get up more than two flights of stairs?				
	- Is there any physical activity that is more difficult than previously?				
**Day-to-day activities and sleep disorders**					
20.24 Increased fatigue					
	- Are there any provoking factors which amplify fatigue?				
	- How many hours a night does the child sleep?				
	- Is the child tired in the morning, despite a good night's sleep?				
	- Are there any day-to-day activities (dressing, washing etc.) that are more difficult now than they were before?				
20.25 Night sweating					
**Neurological sequelae**					
20.26 Loss of taste and/or smell					

Post-COVID-19 symptom assessment questionnaire

20.27 Dizziness				
20.28 Headache				
20.29 Photophobia				
**Cognitive sequelae**				
20.30 Difficulties in concentrating				
20.31 Impaired memory				
20.32 Impaired attention				
20.33 Mood changes				
20.34 Irritability				
20.35 Anxiety/depression				
20.36 Other				

**Other Symptoms**					
20.37 Hair loss					
20.38 Menstrual disturbances					
20.39 Others					
21. How many days has the child not attended school/kindergarten after recovering from COVID-19 because of the above symptoms?					
22. How many times has the child visited the family doctor after recovering from COVID-19?					
23. Has the child visited a specialist after recovering from COVID-19?					
	What kind of specialist? Why?				
**Examination**					
24. What studies have been performed after recovery from COVID-19?					
24.1 Laboratory exams					
	- F.B.C.				

Post-COVID-19 symptom assessment questionnaire

	- CRP/Il-6				
	- Urine analysis				
	- Creatinine/urea				
	- ALAT/ASAT				
	- Antibodies against SARS-CoV-2				
	- Others				
24.2 Imaging exams					
	- X-ray				
	- USG				
	- Others				
